# Combination of aspirin with essential fatty acids is superior to aspirin alone to prevent or ameliorate sepsis or ARDS

**DOI:** 10.1186/s12944-016-0377-2

**Published:** 2016-11-25

**Authors:** Undurti N Das

**Affiliations:** 1 UND Life Sciences, 2020 S 360th St, # K-202, Federal Way, WA 98003 USA; 2 BioScience Research Centre, GVP College of Engineering Campus and Department of Medicine, GVP Hospital, Madhurawada, Visakhapatnam 530 048 India

**Keywords:** Aspirin, Essential fatty acids, Acute respiratory distress syndrome, Sepsis, Lipoxin A4, Resolvin, Protectins, Arachidonic acid, Eicosapentaenoic acid, Docosahexaenoic acid, Nitric oxide, Cyclo-oxygenase, Lipoxygenase

## Abstract

It has been suggested that aspirin may be of benefit in treating sepsis and ARDS in view of its ability to block cyclo-oxygenase-1 (COX-1) and COX-2 activities; inhibit nuclear factor kappa B (NF-κB); enhance the production of endothelial nitric oxide (eNO) and lipoxin A4 (LXA4). Our previous studies revealed that plasma phospholipid content of arachidonic acid (AA) and eicosapentaenoic acid (EPA) is low in patients with sepsis. This implies that beneficial actions of aspirin in sepsis and ARDS is unlikely to be obtained in view of deficiency of AA and EPA, the precursors of LXA4 and resolvins respectively that are potent anti-inflammatory compounds and enhancers of eNO generation. In view of this, I propose that a combination of aspirin and AA and EPA (and possibly, docosahexaenoic acid, DHA) is likely to be superior in the management of sepsis and ARDS compared to aspirin alone. This suggestion is supported by the recent observation that trauma patients with uncomplicated recoveries had higher resolvin pathway gene expression and lower gene expression ratios of leukotriene: resolvin pathways.

## Introduction

ARDS (acute respiratory distress syndrome), sepsis and septic shock can lead to multiorgan dysfunction syndrome (MODS) that cause death among patients in non-coronary critical care units. Several mechanisms contribute to the pathogenesis of MODS. Some of these include: bacterial toxins, inflammatory mediators secreted by neutrophils, macrophages, and T cells; endothelial injury, disturbed homeostasis, and microcirculatory failure. Though the exact pathobiology of ARDS, sepsis and septic shock is not clear, there is significant evidence to suggest that inflammation and disruption of the hemostatic system as a result of platelet-leucocyte interaction and consequent enhanced production of inflammatory cytokines such as interleukin (IL)-1β, IL-8, monocyte chemotactic protein 1 and tumour necrosis factor-alpha (TNF-α) play a major role [1–5]. The septic response is a complex chain of events involving inflammatory and antiinflammatory processes, humoral and cellular reactions and circulatory abnormalities [6–8]. Sepsis and evaluation of its severity is complicated by the highly variable and non-specific nature of the signs and symptoms of sepsis [9].

Sepsis is now defined as evidence of infection plus life-threatening organ dysfunction, clinically characterized by an acute change of 2 points or greater in the SOFA (Sequential [Sepsis-related] Organ Failure Assessment) score. The criteria for septic shock include sepsis with fluid-unresponsive hypotension, serum lactate level greater than 2 mmol/L, and the need for vasopressors to maintain mean arterial pressure of 65 mmHg or greater. The new definitions [10–13] eliminated mention of SIRS (systemic inflammatory response syndrome). Components of SIRS include tachycardia, tachypnea, hyperthermia or hypothermia, and abnormalities in peripheral white blood cell count that are nearly ubiquitous in hospitalized patients and occur in many benign conditions, both related and not related to infection, and thus are not adequately specific for the diagnosis of sepsis. But, it needs to be noted that patients with infections and organ dysfunction are exceptionally heterogeneous in terms of demographic characteristics, underlying conditions, microbiology, and other clinically relevant factors.

The close link among inflammation, disruption of the hemostatic system as a result of platelet-leucocyte interaction and altered production of inflammatory cytokines seen in sepsis led to the suggestion that thrombosis, inflammation and platelets play a significant role in sepsis and hence, anti-platelet medications such as aspirin could be of benefit in sepsis and ARDS [5, 6]. The potential beneficial action of aspirin in ARDS and sepsis has been attributed to its ability to (i) inhibition of COX; (ii) inhibition of nuclear factor kappa B (NFκB); (iii) increase in the production of endothelial nitric oxide (eNO) and inhibition of inducible NO generation; (iv) enhanced production of lipoxin A4 (LXA4) and (v) decrease in the generation of platelet aggregator thromboxane A2 (TXA2) and enhance prostacyclin (PGI2) production [5, 14–20].

The putative beneficial role of aspirin in ARDS and sepsis is supported by the observation that aspirin treatment is effective in murine models of these diseases [5, 21–24]. These results are supported from human observational studies wherein it was noted that aspirin treatment could have decreased risk of developing sepsis and ARDS; reduced severity when they do develop sepsis or ARDS and decreased mortality [5, 24–37]. But these results are not without controversy [28]. These studies are interesting and open a new avenue of managing sepsis and ARDS. But, I suggest that use of aspirin alone may not be adequate in sepsis and ARDS. I propose that a combination of aspirin and unsaturated fatty acids: arachidonic acid (AA, 20:4 n-6), eicosapentaenoic acid (EPA, 20:5 n-3) and docosahexaenoic acid (DHA, 22:6 n-3), the precursors of anti-inflammatory bioactive lipids lipoxin A4 (from AA); resolvins (from EPA and DHA); and protectins and maresins (from DHA) [38–40] is likely to be more suited and beneficial in managing sepsis and ARDS.

## Essential fatty acid (EFA) metabolism is altered in sepsis and ARDS

Previously it was shown that AA metabolism is markedly affected in patients with sepsis [41]. It was reported that AA, AA analogues, and the cyclooxygenase-associated metabolites: prostaglandin E2 (PGE2), 11-hydroxyeicosatetraenoic acid, and thromboxane B2 (TXB2) were identified as differentiating metabolites between septic patients and healthy subjects. AA, its analogues, and PGE2 and TXB2 differed at baseline. It is interesting that the inducibility of AA and the cyclooxygenase metabolites 11-hydroxyeicosatetraenoic and PGE2 were reduced by 80% to 90% in sepsis. Furthermore, the degree of the inducibility was associated with severity of sepsis and clinical outcome. A reduced inducibility of COX-2 but preserved inducibility of mPGES-1 on gene expression level was confirmed in an independent cohort of septic patients compared to healthy controls [41].

**Fig. 1:**
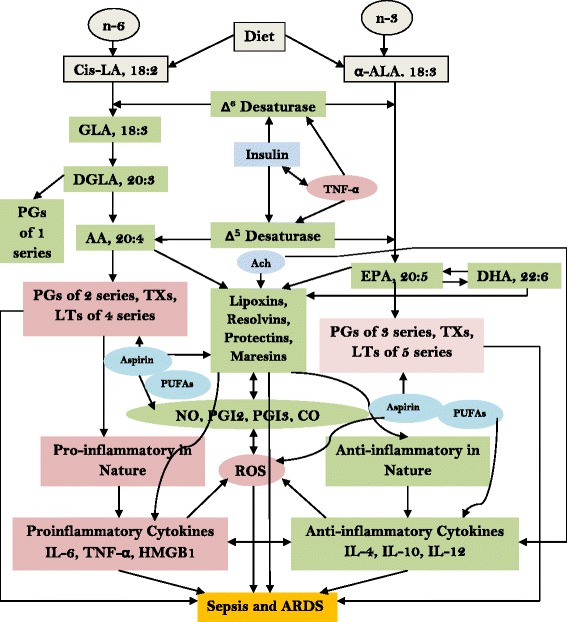
Scheme showing metabolism of EFAs, formation of their metabolites PGs, LTs, TXs, LXA4, resolvins, protectins and maresins and their actions on cytokines, ROS (reactive oxygen species), NO (nitric oxide), carbon monoxide (a product of hemeoxygenase −1) and relevance of aspirin in this chain of events and their role in sepsis and ARDS. Ach = Acetylcholine

Previously, we reported that patients with sepsis showed increased production of superoxide anion and hydrogen peroxide (H_2_O_2_) by their peripheral leukocytes; and enhanced levels of circulating lipid peroxides with a concomitant decrease in plasma phospholipid content of GLA, DGLA, AA of n-6 series and ALA, EPA and DHA of n-3 series (see Table 1) [42]. These findings imply that decreased production of lipoxin A4 (LXA4), resolvins, protectins and maresins may occur due to a deficiency of their precursors (AA, EPA and DHA), which are potent anti-inflammatory bioactive lipids that may lead to perpetuation of the inflammatory process seen in sepsis and ARDS. Though the exact cause for this deficiency of GLA, DGLA, AA, ALA, EPA and DHA is not clear, this may, in part be due to their peroxidation as a result of enhanced generation of free radicals and the ability of TNF-α to suppress the formation of DGLA, AA, EPA and DHA. In an in vitro study, it was noted that TNF-α incubation of HUVEC (human umbilical vascular endothelial cells) caused an EFA deficiency state reminiscent of long-term malnutrition [43]. This interaction between TNF-α and possibly, other pro-inflammatory cytokines and EFA metabolism is significant since several PUFAs (polyunsaturated fatty acids) inhibit TNF-α and IL-6 production and excess free radical generation [38]. Thus, induction of EFA deficiency state by TNF-α in sepsis (and possibly, other conditions in which there is enhanced production of TNF-α occurs) may exacerbate the inflammatory process due to the absence of negative feedback control exerted by PUFAs and their anti-inflammatory metabolites: LXA4, resolvins, protectins and maresins. In such an instance of EFA deficiency states, administration of aspirin may not be able to enhance the formation of LXA4 and aspirin-triggered 15-epi-lipoxin A4 (ATL) (from AA), resolvins (from EPA and DHA), protectins and maresins (from DHA) due to decreased levels of their precursors AA, EPA and DHA. In view of this, it is unlikely that aspirin alone will be of significant benefit in ARDS and sepsis wherein AA, EPA and DHA deficiency is present. This argument is supported by the observation that administration of aspirin produced controversial results in sepsis and ARDS [28]. I propose that co-administration of PUFAs (especially AA, EPA and DHA) along with aspirin is likely to be of more beneficial and rational than administration of aspirin alone. Such a combined provision of PUFAs (especially AA, EPA and DHA) and aspirin is expected to lead to enhanced formation of LXA4, resolvins, protectins, maresins and 15-epi LXA4 (ATL) due to the availability of precursors of these anti-inflammatory bioactive lipids (see Fig. 1).

**Table 1:** Leukocyte free radical generation, plasma lipid peroxides and phospholipid content of unsaturated fatty acids in patients with sepsis

Measurement		Control (*n* = 10)	Sepsis (*n* =14)
μM of formazan (O_2_^−1^)	without PMA	4.5 ± 1.0	6.48 ± 3.0^*^
	with PMA	40.3 ± 9.7	62.2 ± 18^*^
μM of H_2_O_2_	without PMA	4.76 ± 0.56	9.0 ± 4.3^*^
	With PMA	44.2 ± 6.16	70.3 ± 50
nmoles of MDA-eq		1.56 ± 0.36	2.37 ± 0.6^*^
18:3 n-6 (GLA)		0.13 ± 0.09	0.04 ± 0.05^*^
20:3 n-6 (DGLA)		3.2 ± 0.79	0.46 ± 0.54^*^
20:4 n-6 (AA)		8.8 ± 2.0	5.8 ± 1.6^*^
18:3 n-3 (ALA)		0.27 ± 0.12	0.16 ± 0.11^*^
20:5 n-3 (EPA)		0.25 ± 0.26	0.01 ± 0.01^*^
22:6 n-3 (DHA)		1.43 ± 0.43	1.2 ± 1.14

All values are expressed as mean ± SE. *P < 0.05 compared to control. All fatty acids are expressed as % of PL (phospholipid)

## Lipoxins, resolvins and protectins in sepsis and ARDS

The role of PUFAs and their anti-inflammatory bioactive lipids in sepsis is supported by the observation that in the cecal ligation and puncture model of sepsis in rats administration of LXA4 (40 μg/kg intraperitoneally) 5 h after surgery increased survival compared to control [44]. In this study, it was noted that plasma IL-6, monocyte chemotactic protein 1 and IL-10 levels were reduced in addition to reduced phosphorylation of the p65 subunit of NF-κB in LXA4 treated rats suggesting that LXA4 reduced systemic inflammatory events. In addition, LXA4 reduced bacterial load and increased peritoneal macrophage number that accounted for increased bacterial clearance. These results suggested that LXA4 increased survival in sepsis by reducing systemic inflammation [44]. In this context, it is noteworthy that LXA4 but not its stable analog 15-epi-16-(para-fluorophenoxy)-lipoxin A4 methyl ester (when both were given at a dose of 7 μg/kg, i.v.) in the cecal ligation and puncture (CLP) model of sepsis increased 8 day survival despite the fact that both compounds reduced plasma TNF-α and IL-6 concentrations. It was observed that LXA4 analog increased plasma IL-10 concentrations compared to rats given LXA4, while LXA4 reduced blood bacterial load but LXA4 analog did not [45]. These results suggest that endogenous LXA4 is superior in the management of sepsis compared to its more stable and structural analogs that one need to note while developing therapies based on LXA4.

These results are further supported by the observation that resolvin D2, identified in resolving exudates, decreased leukocyte–endothelial interactions in vivo by endothelial-dependent nitric oxide production, and by direct modulation of leukocyte adhesion receptor expression, decreased both local and systemic bacterial burden, excessive cytokine production and neutrophil recruitment, while increasing peritoneal mononuclear cells and macrophage phagocytosis and thus, increased survival from sepsis induced by caecal ligation and puncture and surgery [46]. These beneficial actions of resolvin D2 seem to be mediated by its receptor GPR-18 (G-protein coupled receptor-18) that is expressed on human leukocytes, monocytes and macrophages. The ability of resolvin D2 to limit PMN infiltration, enhance phagocyte clearance of bacteria and accelerate resolution of inflammation was lost in GPR-18-deficient mice emphasizing the role of resolvin D2-GPR-18 resolution axis to control bacterial infections and prevent sepsis and promote organ protection [47]. Thus, LXA4 and resolvin D2 and, possibly, other resolvins, protectins and maresins are of significant benefit in sepsis and ARDS.

In this context, it is noteworthy that the anti-inflammatory and inflammation resolution promotor actions of LXA4, resolvins, protectins and maresins is balanced and antagonized by pro-inflammatory bioactive lipid leukotriene B4 (LTB4). Mice with type 1 diabetes that are susceptible to chronic systemic inflammation and enhanced susceptibility to sepsis showed increased expression of Alox5, which encodes 5-lipoxygenase (5-LOX), and enhanced concentrations of IL-1β in peritoneal macrophages and serum, while treatment with insulin reduced LTB4 concentrations. On the other hand, type 1 diabetic mice lacking 5-LOX or the receptor for LTB4 produced less IL-1β, survived polymicrobial sepsis, and had decreased bacterial counts [48]. These results [44–48] imply that under normal physiological condition there is a balance maintained between pro- and anti-inflammatory bioactive lipids (LTB4 *vs* LXA4/resolvins/protectins/maresins) to limit inflammation, regulate production of various cytokines, control bacterial proliferation and their ability to produce inflammation and ultimately control, prevent and help in the recovery from sepsis and ARDS as proposed previously [49, 50].

Pneumosepsis induced in mice by *Klebsiella pneumoniae* produced an early increase in LXA4 and its receptor FPR2/ALX lung expression, local and systemic infection and inflammation, and mortality, suggesting that excess production of LXA4 may also be harmful. On the other hand, treatment with LXA4 antagonist in early sepsis increased leukocyte migration to the focus, and reduced bacterial load and dissemination and improved survival. In contrast, it was also noted that treatment with LXA4 in early sepsis decreased cell migration and worsened the infection. On the other hand, in late sepsis, LXA4 improved the survival rate by reducing the excessive inflammatory response. Thus, LXA4 and its receptor FPR2/ALX seem to have a dual role in sepsis and its beneficial or harmful effects are critically dependent on the time [51]. It is likely that resolvins, protectins and maresins also may have actions similar to LXA4 in sepsis. These results emphasize the importance of timing in the use of proresolving anti-inflammatory bioactive lipids in the management of sepsis and ARDS.

The ability of insulin to suppress LTB4 and IL-1β production is in tune with the previous suggestion that insulin has anti-inflammatory actions and its infusion may be of benefit in sepsis and ARDS [52–55]. Furthermore, insulin is known to activate Δ^6^ and Δ^5^ desaturases that are needed for the conversion of EFAs: linoleic acid (LA, 18:2 n-6) and α-linolenic acid (ALA, 18:3 n-3) to their long-chain metabolites GLA, DGLA, AA, and EPA and DHA respectively [20]. Thus, a co-ordinated and negative and positive feedback regulatory action among insulin, Δ^6^ and Δ^5^ desaturases, various PUFAs and their pro- and anti-inflammatory bioactive lipids and cytokines is needed to control infection and recover from sepsis and ARDS.

## PUFAs, LXA4, resolvins, protectins and maresins have antibiotic-like actions and modulate phagocytosis

It is interesting to note that PUFAs: LA, ALA, GLA, AA, EPA and DHA have anti-bacterial actions [56, 57]. Recent studies showed that GLA and AA enhance the activities of ceftazidime and amikacin on multidrug-resistant *Pseudomonas aeruginosa* and *Escherichia coli* both *in vitro* and *in vivo*. PUFAs are present in all cell membranes and released in response to many stimuli especially on exposure to endotoxin (lipopolysaccharide: LPS) of bacteria due to activation of phospholipase A2 (PLA2). These results suggest that endogenous unsaturated fatty acids may function as natural antibiotics. This raises the intriguing possibility that a deficiency of these endogenous unsaturated fatty acids (especially of GLA and AA and EPA) may predispose an individual to various infections. When the susceptibility of 242 strains of *Staphylococcus aureus* and 117 strains of *streptococci* of groups A, B, C, and G to various fatty acids were studied, it was found that GLA, AA, EPA and DHA are effective against them [58–64]. Similar, if not identical, anti-bacterial activity was shown to be possessed by resolvins and protectins which enhanced antibiotic ciprofloxacin therapeutic actions and enhanced vancomycin clearance of *Staphylococcus aureus* [65]. But, it is not yet clear whether resolvins, protectins, maresins and LXA4 can directly inhibit bacterial growth similar to PUFAs.

It is well known that hyperactivation of neutrophils that is seen in early phase of sepsis leads to tissue injury. In contrast, neutrophil dysregulation in the form of reduced levels of migration, decreased apoptosis and inadequate phagocytosis that occurs in the later part of sepsis impairs host’s ability to clear infection. A recent study [66] showed that in the cecal ligation and puncture rat model of sepsis, LXA4 given after 1 h reduced blood bacterial load at 24 h, decreased neutrophil migration to the peritoneum but augmented blood neutrophil phagocytic ability and promoted apoptosis without affecting free radical production. In addition, LXA4 increased peritoneal neutrophil phagocytic ability without affecting apoptosis or free radical production suggesting that effects of LXA4 were compartment specific. Incubation ex vivo LXA4 (1 nM) increased phagocytosis in blood neutrophils without affecting apoptosis or free radical production. These results suggest that LXA4 increased neutrophil bacteria clearing function without excessive free radical production, a beneficial action needed in sepsis. Similar results were also obtained in acute lung injury (ALI).

ALI is a severe illness with excess mortality and no specific therapy and is often seen in sepsis that leads to ARDS. In its early exudative phase, neutrophil activation and accumulation in the lung lead to hypoxemia, widespread tissue damage, and respiratory failure (ARDS). In a spontaneously resolving experimental murine model of ALI, inhibition of COX-2 blocked resolution of ALI. Both LXA4 and 15-epi-LXA4 produced dramatic protection from ALI, indicating that LXA4 has a protective role in ALI and ARDS and COX-2 expression is needed for this purpose [67]. These results are supported by the observation that inhalation of LPS (lipopolysaccharide)-induced increased inflammatory cell counts, TNF-α and protein concentration in bronchoalveolar lavage fluid and lung injury and edema can be prevented by aspirin triggered lipoxin A4 (ATL, 15-epi-16-parafluorophenoxy lipoxin A4), a stable analogue of LXA4 [68]. In addition, ATL promoted the formation of heme oxygenase-1 in the lung tissues. Heme oxygenase-1 activity, which offers protection against prooxidant insults [69], was also increased in the lung tissues after ATL treatment, demonstrating that ATL significantly reduces LPS-induced acute lung injury (and ARDS) in mice. In this context, it is interesting to note that activation of the efferent vagus nerve inhibits the release of TNF-α and attenuates the development of endotoxin-induced shock in rodents [70]. Stimulation of the efferent vagus nerve activates the release of acetylcholine. There is indirect evidence to suggest that acetylcholine, an anti-inflammatory molecule, augments LXA4 production [71]. Thus, there appears to be a link between neurohumoral pathway and anti-inflammatory lipid mediators such as LXA4, resolvins and protectins.

## Clinical studies

Despite many *in vitro* and animal studies, there is scarcity of information on the role of LXA4, resolvins, protectins and maresins in patients with sepsis and ARDS. In a recent study, it was noted that the ratio of expression between endogenous proinflammatory (leukotrienes) and proresolving (resolvins) lipid mediator pathway genes was significantly higher in patients with prolonged, complicated recovery after blunt trauma, suggesting that patients with complicated recoveries have dysregulated lipid mediator signaling [72]. It is likely that resolvins, protectins and maresins also may have actions similar to LXA4 in sepsis. These results emphasize the importance of timing in the use of proresolving anti-inflammatory bioactive lipids in the management of sepsis and ARDS.

Examination of 7,945 intensive care unit admissions showed a strong association between acetyl salicylic acid and survival in intensive care unit SIRS and sepsis patients. These results led to the suggestion that low doses of acetyl salicylic acid, acting through 15-epi-lipoxin A4, are responsible for its beneficial action in reducing the severity of SIRS and reduced mortality [30].

Despite many studies in experimental animals showing beneficial action of PUFAs especially of EPA and DHA (given in the form of fish oil in majority of the studies) [73–75] in sepsis, these beneficial actions were not uniformly seen in patients [76, 77]. But some clinical studies did show that PUFAs may be beneficial in patients with sepsis [78–84]. These contradictory results [73–84] led ‘Surviving Sepsis Campaign: International Guidelines for Management of Severe Sepsis and Septic Shock’ to recommend that there is no sufficient evidence for the use of immunomodulating nutritional supplements such as PUFAs in sepsis [85], though larger trials are ongoing. Based on these evidences, it is suggested that better defined clinical studies with different combinations of PUFAs (example: EPA ± DHA ± GLA ± AA ± DPA) using different doses of fatty acids for various periods of time (example: few hours to days to weeks) are needed to determine the efficacy of PUFAs in sepsis and ARDS.

## Conclusions

Obviously sepsis and ARDS are as a result of multiple causes with multiple mediators involved in their pathogenesis. Though the concept of use of aspirin in sepsis and ARDS appears interesting and novel, the presence of EFA deficiency and an imbalance between pro and anti-inflammatory molecules suggests that perhaps, use of GLA, AA, EPA and DHA along with aspirin to enhance the production of anti-inflammatory LXA4, resolvins, protectins and maresins is more prudent. A recent multicentre study in patients undergoing mechanical ventilation with severe sepsis and septic shock reported a 19.4% reduction in the absolute risk of mortality, improved oxygenation, more days free from mechanical ventilation, decreased stay at the ICU and less development of new organic dysfunctions in the group that received EPA, LA and anti-oxidants [86]. A more recent multicenter study showed a significant decrease in the mean length of stay in the ICU without affecting mortality or infectious complications in the intention to treat analysis in those who received EPA and GLA infusions [76]. It was reported that inclusion of fish oil in parenteral nutrition provided to septic ICU patients increased plasma EPA, suppressed inflammatory cytokine concentrations and improves gas exchange [87]. These changes are associated with a tendency towards shorter length of hospital stay. It is possible that had the investigators included aspirin and DHA and AA in these in the infusions, perhaps the results would have been more encouraging.

In this context, it is worthwhile to measure plasma levels of lipid peroxides, various cytokines, PUFAs, LXA4, resolvins, protectins, maresins, LTB4 and correlate their concentrations to the severity and prognosis of sepsis and ARDS especially prior to embarking on the trial of aspirin with various PUFAs in order to know the dynamics of their changes in these patients as suggested previously [50]. Such a knowledge will gives us a better handle as to how much and when to administer aspirin, various PUFAs and possibly, LXA4, resolvins, protectins and maresins to those with sepsis and ARDS.
